# A Case Series and Technical Note on Endoluminal Vacuum Therapy via Pull-Through Technique for Leakage after Metabolic Bariatric Surgery

**DOI:** 10.1007/s11695-026-08563-y

**Published:** 2026-03-24

**Authors:** Lisa Gensthaler, Max Stauffer, Julia Jedamzik, Christoph Bichler, Larissa Nixdorf, Paula Richwien, Felix B. Langer, Gerhard Prager, Daniel M. Felsenreich

**Affiliations:** Department of General Surgery, Medical University of Vienna, Vienna, Austria

**Keywords:** Bariatric surgery, Revisional surgery, Endoluminal Vacuum Therapy, Pull-through technique

## Abstract

**Background:**

Endoluminal vacuum therapy (EVT, Eso-SPONGE^®^) is an effective treatment for leaks following metabolic-bariatric surgery (MBS), used for staple line- or anastomotic leakage. While EVT is conventionally placed using an overtube, the pull-through technique is a convenient alternative for hardly accessible dehiscence or large wound cavity, allowing precise sponge placement for optimal drainage. This analysis provides a technical note and evaluates pull-through EVT’s efficacy in MBS patients.

**Material & methods:**

All patients treated for leaks after MBS with EVT pull-through technique between 01/2018 and 06/2025 at the Department of General Surgery, Medical University of Vienna (MUV), were included in this retrospective analysis. Statistical analyses were performed descriptively.

**Results:**

7 patients were treated with EVT via pull-through technique. In 5/7 cases (71.4%), index surgery was a primary MBS and acute leaks occurred in 6/7 cases (85.7%). All patients received surgical reintervention, targeted drainage and EVT (7/7; 100%). Self-expanding metal stent was used in 4/7 cases (57.1%) additionally. EVT was changed 9.6 times (3–33) with EVT duration of 30.8 days (11–97). No severe device-related complications were observed. Successful leak closure with EVT was achieved in 85.7%.

**Conclusion:**

EVT via pull-through technique is effective and feasible for managing leaks after MBS when targeted drainage is available. It allows precise EVT placement and enhanced tissue healing, potentially reducing necessity for further reoperation in critically ill patients.

## Key points

1. Endoluminal vacuum therapy via pull-through technique is an efficient and minimally invasive therapy for postoperative leakage after MBS.

2. Pull-through EVT can be applied in patients with hardly accessible dehiscence and big wound cavity, if a targeted drainage is in place.

3. In all cases, a combination of surgical reintervention and EVT placement was necessary.

4. Pull-through EVT should be established in daily clinical practice, supporting clinical outcome of critically ill MBS patients.

## Background

Metabolic/bariatric surgery (MBS) is safe and a highly effective treatment for obesity [1–3]. Nevertheless, complications such as anastomotic- or staple-line leakage (AL or SLL) are described in 1.5% to 5.6% [4–6]. Prompt, individualized complication management is essential, particularly in patients with obesity-related comorbidities such as cardiovascular disease, obstructive sleep apnea or type 2 diabetes mellitus. Following the therapeutic approach in upper GI and colorectal surgery [7–10], endoluminal vacuum therapy (EVT) is an effective, minimally invasive alternative to surgical reintervention, reducing morbidity and mortality in critically ill patients, especially in acute (day 0–7) or early leaks (week 1–6) [11, 12]. At the Medical University of Vienna (MUV), the largest national MBS center, experience and outcomes with EVT in case of postoperative leakage after MBS have been published recently by Gensthaler et al. [11]. Typically, the Eso-SPONGE^®^ is placed via an overtube, but in complex cases, when a big abscess or wound cavity is present, this technique meets its limits. The pull-through technique is an elegant alternative for proper intracavitary EVT placement and adequate drainage. This small case series and technical note describe the EVT pull-through technique after – or instead of – revisional MBS, focusing on patient selection, stepwise guidance for application, perioperative management and outcome. It represents a detailed sub-analysis based on the previously published manuscript by Gensthaler et al., which evaluated the effectiveness and clinical feasibility of all patients with EVT after MBS [11].

## Material & Methods

All patients, treated with pull-through EVT due to AL or SLL after MBS at the MUV, a high-volume MBS center, between 01/2018 and 06/2025 were included in this retrospective analysis. It was approved by the local ethics committee (1279/2022) and data were collected retrospectively and pseudonymized. Patients undergoing surgery at the MUV and those, referred from other bariatric centers for leak treatment after MBS were included. All re-interventions at the MUV were performed by the same specialized surgical MBS team.

## Patient and Clinical Characteristics

Baseline data included age, sex, BMI, comorbidities, medical history, nicotine- and alcohol consumption. Index surgeries were primary MBS procedures or reinterventions for long-term complications such as internal hernia and marginal ulcer. Surgical procedures were classified as Sleeve Gastrectomy (SG), Gastric Bypass (GB) such as One-Anastomosis Gastric Bypass (OAGB), Roux-en-Y Gastric Bypass (RYGB), Single-Anastomosis-Duodeno-Ileal Bypass with Sleeve Gastrectomy (SADI-S) or revisional surgery (RS). Site of primary surgery was the MUV or an external MBS center. ICU (intensive care unit) admission, length of stay, complications and mortality during follow-up was evaluated. Time from index surgery to onset of symptoms, to leak diagnosis, to first surgical intervention and to implementation of EVT were assessed.

## Diagnostic Management of Leakage and Perioperative Care

Leakage after MBS is diagnosed based on clinical evaluation and parameters such as tachycardia, hypotension, fever, pain or elevated infection parameters and confirmed via contrast swallow X-ray or swallow-CT scans with water soluble contrast medium. Leaks were classified by the classification of Rosenthal et al. [13]. Endoscopy (Olympus HQ190 TEE) can be applied as diagnostic tool for leakage at primary or surgical intervention with an intraoperative air-leak test, assessment the integrity of the staple line or anastomosis and exploring the wound cavity or dehiscence during surgical reintervention. Proper drainage during surgical reintervention is necessary for adequate drainage and obligatory for future EVT placement via pull-through technique.

Therapy was chosen individually, depending on patients’ clinical condition and location/extent of the dehiscence. At external centers, surgical reintervention and SEMS were performed instead of EVT preferably. EVT, effective for sufficient granulation in revisional MBS [11] is conventionally placed using the “overtube-technique”. By using the pull-through technique, even more precise placement of the sponge into the leakage or hardly accessible wound cavity is possible. During EVT, oral food cessation is obligatory, and parenteral nutrition will be implemented, depending on caloric demand. To ensure excellent quality, surgical reintervention and endoscopic interventions such as EVT placement, especially if the pull-through technique is used, should always be performed by the same group of specialized MBS surgeons. Therapeutic success or efficacy of EVT via pull-through technique was defined as complete leak closure.

### Surgical Technique and EVT Placement Via Pull-through Technique

#### Preparation and Positioning

EVT placement is always performed in the operating theatre with short endotracheal intubation for airway safety. Patients are positioned supine on the operating table to facilitate optimal endoscopic access. Next to a flexible endoscope (Olympus HQ190 TEE) and the Eso-SPONGE^®^ equipment, a radiologic guidewire, a non-resorbable suture of minimum 90 cm length and a C-armed X-ray unit must be prepared.

#### EVT Placement Via Pull-through Technique

**Fig. 1:**
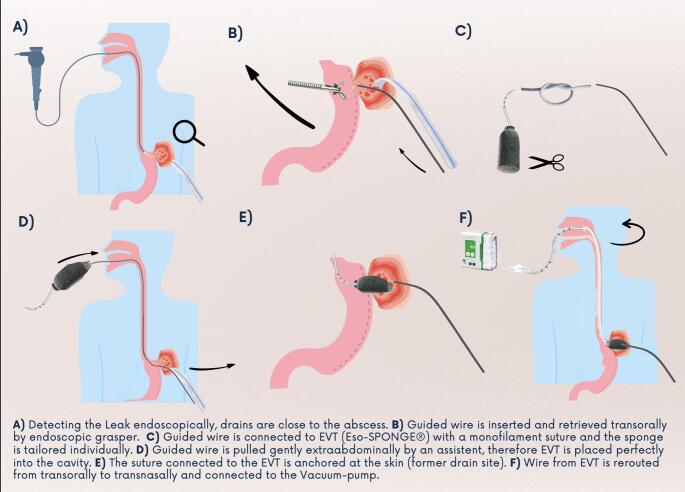
Stepwise Technique of Pull-Through EVT: Graphical Illustration

**Fig. 2:**
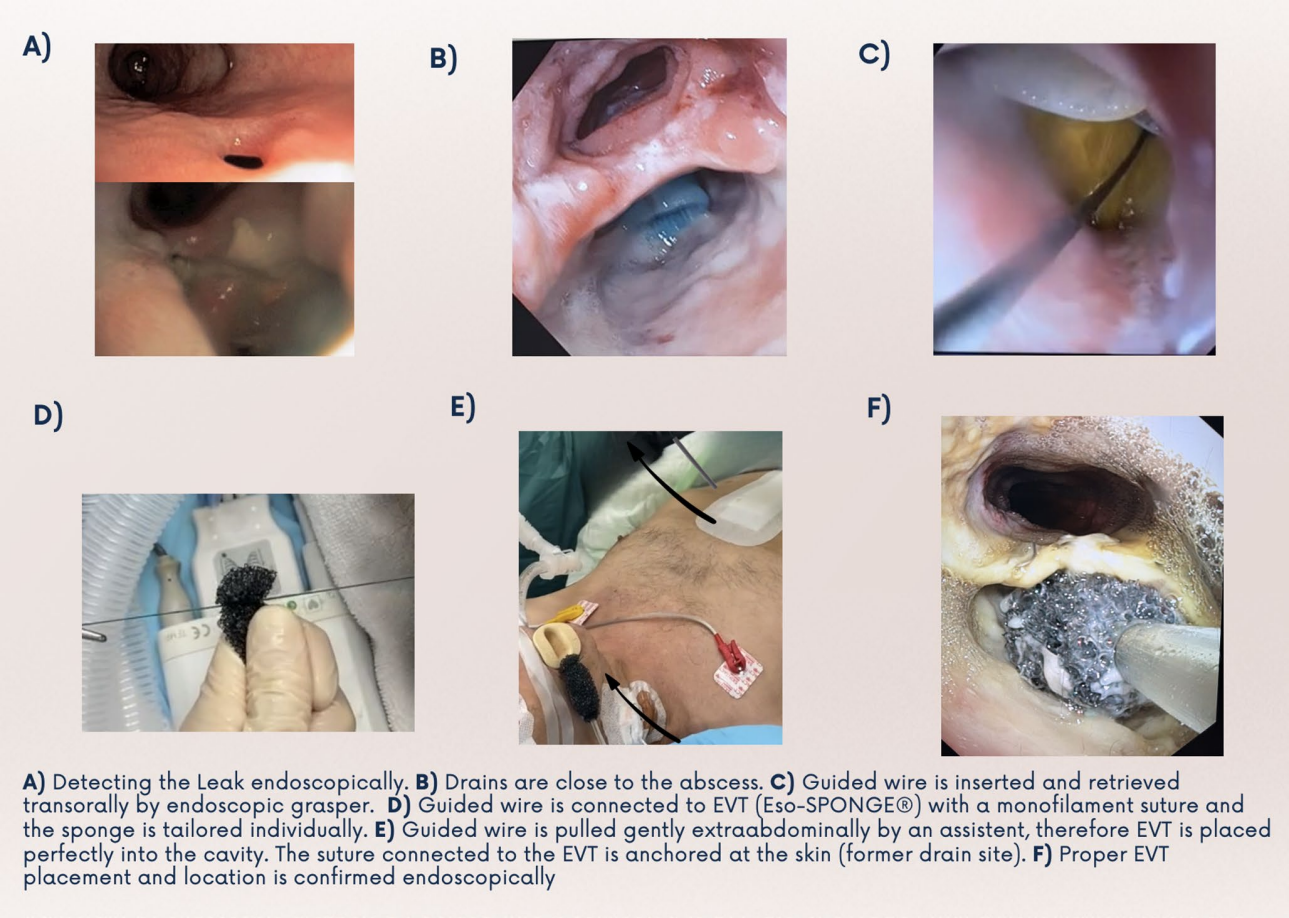
Stepwise Technique of Pull-Through EVT: Intraoperative Visualization

The endoscope is inserted orally and maneuvered to the site of dehiscence, where the previously placed drains are visualized (Figs. 1 and 2). Under radiologic surveillance, the guidewire is inserted alongside/through pre-existing drains from extra- to intraabdominally. As soon as endoscopically visible, the guidewire is grasped and retrieved trans-orally under endoscopic visualization. Alternatively, drains visualized endoscopically can be grasped and retrieved extra-abdominally. A non-absorbable suture is then attached, and both are retrieved trans-orally. Afterwards, guidewire and Eso-SPONGE^®^ are connected precisely by knots of a non-absorbable suture. The Guidewire can then be retrieved under radiologic surveillance until the suture appears extra-abdominally next to the drains. Therefore, precise placement of EVT within the leakage or cavity is enabled. It’s important not to pull against resistance to avoid tissue injury. The suction tube is rerouted trans-nasally and connected with the vacuum pump. Continuous suction of −60 to −80 mmHg at slurp mode is applied to ensure sufficient drainage. The residual suture is carefully fixed at the abdominal wall and due to the remaining drains, proper drainage and sufficient EVT functioning can be observed in case of high-volume secretion. A stepwise illustration of EVT placement can be observed in Figs. 1 and 2. If an abscess is present, a CT guided drainage can be placed allowing EVT placement with a guidewire, pushed through the drainage channel.

#### EVT Replacement

EVT sponges are replaced every 3–4 days, using the residual suture to guide transoral replacement. It is obligatory to watch the remaining suture at the site of drainage carefully to maintain the pull-through process. Once the sponge is retrieved, trans-orally, the suture must be reconnected to a fresh sponge. This procedure always includes endoscopic evaluation of the wound cavity and granulation progress. For replacement of the sponge, the thread must be pulled gently to place the fresh Eso-SPONGE^®^ precisely into the wound cavity. The EVT sponge is individually tailored to the size of the wound cavity and gradually reduced as therapy progresses. Once the leak is reduced to a fistula, a perforated sheet can be wrapped around a 16 french nasogastric tube and placed via pull through technique in the most precise way, promoting final granulation.

### Statistical Analysis

Patient population was characterized by descriptive statistics. Categorical variables are presented as numbers (n) and proportions (%), continuous variables as median (range) or mean (± SD). Analyses were performed using SPSS v30 for Mac (IBM, Armonk, New York, USA).

## Results

### Patient and Clinical Characteristics

Due to leakage after MBS, 7 patients were treated with EVT placed via pull-through technique during 2018–2025. 4 (*n* = 4/7; 57.1%) patients were female and mean age was 47.4 (22–67) years. Mean BMI at index surgery was 43.8 kg/m^2^ (24–60 kg/m^2^). Patients had various obesity-associated comorbidities such as arterial hypertension (*n* = 2/7; 28.6%) and diabetes mellitus (DM II, *n* = 3/7; 42.9%). Pathologic nicotine- and alcohol consumption was reported by 5 (*n* = 5/7; 71.4%) and one patient (*n* = 1/7; 14.3%). In 5 cases (*n* = 5/7; 71.4%), index surgery was a primary MBS intervention. One patient underwent SG (*n* = 1/7; 14.3%), in four cases (*n* = 4/7; 57.1%) a GB (1 OAGB, 2 RYGB, 1 SADI-S) was performed. In these two patients receiving surgical reintervention, an anastomotic revision was performed due to a perforated anastomotic ulcer and an internal hernia at outward MBS centers. Four patients (*n* = 4/7; 57.1%) underwent index surgery at the MUV, while 3 (*n* = 3/7; 42.9%) were referred from external MBS centers due to severe postoperative complications, requiring ICU admission. Detailed information is provided in Table 1.

Mean time from index surgery to leak diagnosis was six days [1–13]. Six patients (*n* = 6/7; 85.7%) developed an acute leak (day 0–7) after index surgery, one patient an early leak at 13 days (*n* = 1/7; 14.3%). No late or chronic leaks were observed. Contrast-swallow CT-scan or swallow-X-ray detected leaks in 6 cases (*n* = 6/7; 85.7%), while one patient underwent immediate reoperation. Endoscopy was performed in all patients (*n* = 7/7) and detected all leaks (100%) during surgical reintervention in the air-leak bubble test. In three cases (*n* = 3/7; 42.9%; 1 SG, 1 SADI-S, 1 YRGB), the dehiscence was localized at the upper staple line or esophagogastric junction (EGJ) and in four cases (*n* = 4/7; 57.1%) at the gastro-jejunostomy. Two patients developed acute liver dysfunction and one patient acute kidney failure with necessity for hemodialysis. One patient developed short bowel syndrome after multiple surgical reinterventions. Additional information is shown in Table 1.

**Table 1:** Patients’ and clinical characteristics

Patients' and clinical characteristics *n*	(% or IQR)
**Sex** Male Female	3 (42.9%) 4 (57.1%)
**Age (years**, **IQR)**	47.4 (22–67)
**BMI (kg/m** ^**2**^ **) **	43.8 kg/m^2^ (24–60)
**Comorbidities at the time of admission** Type 2 diabetes Arterial hypertension Nicotine abuse Pathologic alcohol consumption	3 (42.9%) 2 (28.6%) 5 (71.4%) 1 (14.3%)
**Index surgery** Primary Secondary	5 (71.4%) 2 (28.6%)
**Surgical technique** Sleeve gastrectomy RYGB OAGB SADI-S Revisional surgery	1 (14.3%) 2 (28.6%) 1 (14.3%) 1 (14.3%) 2 (28.6%)
**Location of primary surgery** Medical University of Vienna (MUV) Outward bariatric center	4 (57.1%) 3 (42.9%)
**Interval MBS - symptoms (days)**	5.2 (1–12)
**Time of diagnosis** ^**§**^ Acute (day 0–7) Early (week 1–6)	6 (85.7%) 1 (14.3%)
**Detection of leak in CT-scan**	6 (85.7%)
**Detection of leak in endoscopy**	7 (100%)
**Localisation of dehiscence** Staple line/EGJ (SLL)* Gastro-jejunostomy (AL)	3 (42.9%) 4 (57.1%)
**Accompanied complications during EVT** Liver dysfunction Acute Kidney Failure Central pulmonary embolysm	2 (28.6%) 1 (14.3%) 1 (14.3%)

*IQR:* Interquartile-Range, *BMI*: Body Mass Index, *RYGB:* Roux-en-Y Gastric Bypass, *OAGB:* One-Anastomosis Gastric Bypass, *SADI-S:* Single-Anastomosis-Duodeno-Ileale Bypass with Sleeve Gastrectomy, *EGJ*: Esophago-Gastric Junction, Range days: Median (Minimum-Maximum)

*Most upper part of the staple line in patients after Sleeve Gastrectomy, SADI-S, pouch revision

^**§**^ According to the classification by Rosenthal et al. [13]

### Therapeutic Procedure

Mean time from index surgery to first surgical reintervention was 6.3 days (0–15). Time from leakage diagnosis to EVT implementation 17.1 days (0–39) due to primarily surgical reintervention or SEMS in some cases. All patients received a combination of surgical reintervention with targeted drainage, endoscopy, overstitching the leak, and EVT placement via pull-through (*n* = 7/7; 100.0%). In those, who received MBS elsewhere (*n* = 3/7; 42.8%), first surgical reintervention was performed there.

EVT was changed a median of 9.6 times (3–33) every 3–4 days at −60 to −80mmHg with a mean duration of 30.8 days (11–97). No severe device-related complications occurred. Nevertheless, one EVT dislocation and one interruption due to non-compliance were observed. Four patients (*n* = 4/7; 57.1%) underwent additional SEMS placement, two (*n* = 2/3; 66.6%) of them at outward centers prior to transfer. One patient developed a blow-out fistula after SADI-S and received SEMS at the MUV prior to EVT, in one case SEMS was applied intermittent to EVT due to non-compliance and necessity for a pause of EVT. ICU care was necessary for all patients (*n* = 7/7; 100%) with a mean length of stay of 43.9 days (17–99). Mean hospital stay was 118.4 days (21–217). All patients received intravenous nutrition and antimicrobial therapy.

### Follow-up and Outcome

Median follow up of this study was 27.2 months (0.7–71.6). One patient (1/7;14.3%) developed dysphagia, requiring endoscopic dilatation. Three patients (*n* = 3/7; 42.9%) passed away during follow-up: One sustained a central pulmonary embolism during ICU admission 21 days after index surgery, one due to postoperatively developed short bowel syndrome and electrolyte deficiency after 944 days, and one from bacterial uremia, sepsis and kidney failure 339 days after index surgery. Complete leak closure (e.g. therapeutic success) was achieved in 6 patients (*n* = 6/7; 85.7%). Detailed information is provided in Table 2.

**Table 2:** Therapeutic procedure and outcome

Therapeutic procedure & outcome *n*	(%, min-max)
**Therapeutic strategy** Standalone EVT Combination of diagnostic laparoscopy + EVT Intermittent SEMS therapy Prior to EVT (outward MBS clinic)	0 (0.0%) 7 (100.0%) 1 (14.3%) 3 (42.9%)
**Interval index surgery-first reintervention(days)**	6.3 (0–15)
**Interval diagnosis - EVT application (days)**	17.1 (0–39)
**Frequency EVT replacements (n)**	9.6 (3–33)
**Mean EVT dwell time (days)**	30.8 (11–97)
**EVT Complications ** Dislocation Discontinuation due to incompliance	1 (14.3%) 1 (14.3%)
**Admission ICU**	7 (100.0%)
**Length of ICU stay (days)**	43.9 (17–99)
**Lenght of hospital stay (days)**	118.4 (21–217)
**Follow up (median)**	828.0 (21–2176)
**Follow – Up (complications/side effects)** Dysphagia with pneumatic dilatation Short Bowel Syndrome	2 (28.6%) 1 (14.3%)
**Mortality** <90 days after index surgery >90 days after index surgery	1 (14.3%) 2 (28.6%)
**Success with EVT**	6 (95.2%)

*EVT:* Endoluminal vacuum therapy, *SEMS:* Self expanding metal stent, *ICU:* Intensive Care Unit, Range days: Median (Minimum-Maximum)

## Discussion

This retrospective, single-center case series evaluated complex MBS complications’ management with EVT via pull-through technique and an alternative to common EVT placement. Over 7 years (01/2018-06/2025), 7 patients were treated with EVT for SLL or AL at the MUV, achieving successful leak closure in 6/7 (85.7%) cases. This is a sub-analysis of Gensthaler et al., who analyzed all patients, receiving EVT after MBS and observed a 95.2% success rate with EVT in a cohort of 21 patients [11], supporting EVT via pull-through technique as a favorable alternative to surgical reintervention or SEMS [8–10, 14]. It can be easily applied during or after surgical reintervention, if a targeted drainage is in place and close to the dehiscence. It’s safe and minimally invasive for managing hardly accessible leaks in in high-risk patients, allowing precise intracavitary placement and optimal drainage at anatomically challenging locations. It seems to be more effective in acute or early leakage, but this assumption may be influenced by the moment of diagnosis in this case series. Nevertheless, early detection, prompt intervention and opportunity for ICU admission remain essential and treatment of these complex patients should be centralized in specialized, high-volume MBS centers.

### Patients’ Characteristics

Patients in this case series represent a high-risk population regarding morbidity and mortality with severe obesity (mean BMI 43.8 kg/m²) and multiple obesity-associated comorbidities. All patients required ICU care due to sepsis, organ dysfunction or invasive monitoring and treatment. Similar to the results of this study, Kollmann et al. [15] described a 100% success of EVT for MBS leaks in 17 patients, emphasizing the feasibility of EVT in critically ill and complex patients and importance of specialized, high-volume MBS centers with ICU capability for managing complex postoperative complications.

### Clinical Presentation and Diagnosis

Leaks mainly occurred within 1–6 days after index surgery (*n* = 6/7; 85.7%), with radiologic imaging showing high diagnostic reliability [16]. Delayed diagnosis was often associated with local peritonitis or abscess, emphasizing early diagnostic re-laparoscopy, when complication is suspected [10, 17].Therefore, careful postoperative monitoring and clinical follow-up is essential to detect complications early and prevent delay in diagnosis and therapy.

### Therapeutic Procedure

Median duration of pull-through EVT was similar to a meta-analysis by Intriago et al., but longer than in the publication by Gensthaler et al. with duration of 30.8 vs. 25.7 days and 9.6 vs. 6 device exchanges [18], suggesting that patients with pull-through EVT suffered from more complex and severe dehiscence [11]. The main therapeutic approach consisted of surgical reintervention combined with pull-through EVT placement at the MUV. In the larger EVT cohort published by Gensthaler et al., endoscopic treatment alone was sufficient in selected cases and should be considered on an individual basis. Main advantages include minimally invasive placement and ability to promote epithelialization in hardly accessible locations, particularly relevant in critically ill patients. Nevertheless, at outward MBS centers, SEMS was preferred (*n* = 2/3; 42.9%) and possibly delayed sufficient therapeutic approach, highlighting the importance for training to promote EVT usage on a low threshold in daily clinical practice [8]. Prompt EVT initiation, ideally at index endoscopy, can potentially reduce patient’s morbidity [11, 15, 19].

### Outcome

Despite effective leak control, overall morbidity and mortality remained high, reflecting the fragility of this cohort. All patients required ICU care (mean 43.9 days) and prolonged hospitalization (118.4 days), Table 1. One patient died at ICU due to pulmonary embolism (PE) and two during follow-up due to short bowel syndrome and acute kidney failure - none were directly linked to EVT but rather patient’s fragile/critically ill condition. Complete leak closure was achieved in 85.7%, confirming EVT’s efficacy in critically ill patients. Similar outcomes with 100% success were reported by Engelke et al. (2025) with pull-through EVT [19].

### Strengths and Limitations

All procedures were performed by the same specialized surgical team, ensuring therapeutic consistency. EVT is safe, minimally invasive and a favorable alternative to surgery, with reduced morbidity and effective local leak control [18]. Optimized management at ICU/hospital stay with perioperative nutrition and physiotherapy optimizes outcome.

Limitations include the small, heterogeneous and multimorbid sample size, requiring individualized treatment approaches. Leak orifices and cavity size were not assessed routinely. EVT requires prolonged therapy and therefore patient compliance. A delay from diagnosis to EVT implementation was observed, suggesting earlier EVT implementation in these critically ill patients. Due to combined use of SEMS in some cases, it is difficult to attribute therapeutic success solely to pull-through EVT and has to be interpreted with caution.

## Conclusion

EVT via pull-through technique is safe, minimally invasive, and effective approach for SLL and AL after MBS in critically ill patients. It allows precise targeted drainage in challenging anatomical locations, promotes tissue epithelialization and may reduce the need for surgical reintervention. Early recognition, individualized therapy and multidisciplinary treatment should be conducted at specialized, high-volume MBS centers, supporting EVT as effective tool in complex MBS cases.

## Data Availability

No datasets were generated or analysed during the current study.

## Abbreviations

AL: Anastomotic leakage
BMI: Body Mass Index (kg/m^2^)
CT: Computed tomography
EVT: Endoscopic vacuum therapy
GB: Gastric Bypass
GI: Gastrointestinal
ICU: Intensive Care Unit
MBS: Metabolic/Bariatric Surgery
MUV: Medical University of Vienna
NaCl: Sodium chloride
OAGB: One-Anastomosis gastric bypass
RWG: Recurrent weight gain
RYGB: Roux-en-Y gastric bypass
SADI-S: Single-Anastomosis-Duodeno-Ileale Bypass with Sleeve Gastrectomy
SEMS: Self-expanding metal stent
SG: Sleeve gastrectomy
SLL: Staple-line leakage
